# No evidence of immediate fitness benefits of within-season divorce in monogamous birds

**DOI:** 10.1098/rsbl.2021.0671

**Published:** 2022-05-11

**Authors:** Antica Culina, Lyanne Brouwer

**Affiliations:** ^1^ Netherlands Institute of Ecology, NIOO-KNAW, Wageningen, The Netherlands; ^2^ Ruder Boskovic Institute, Zagreb, Croatia; ^3^ College of Science and Engineering, James Cook University, Townsville, Queensland 4811, Australia; ^4^ Division of Ecology and Evolution of Research School of Biology, The Australian National University, Canberra, Australian Capital Territory 2601, Australia

**Keywords:** within-season divorce, birds, monogamy, meta-analysis, extra-pair paternity

## Abstract

Individuals of socially monogamous species can correct for suboptimal partnerships via two secondary mating strategies: divorce and extra-pair mating, with the former potentially providing both genetic and social benefits. Divorcing between breeding seasons has been shown to be generally adaptive behaviour across monogamous birds. Interestingly, some pairs also divorce during the breeding season, when constraints on finding a new partner are stronger. Despite being important for a comprehensive understanding of the evolution of social monogamy, whether within-season divorce is adaptive and how it relates to extra-pair mating remains unknown. Here, we meta-analysed 90 effect sizes on within-season divorce and breeding success, extracted from 31 studies on 24 species. We found no evidence that within-season divorce is adaptive for breeding success. However, the large heterogeneity of effect sizes and strong phylogenetic signal suggest social and environmental factors—which have rarely been considered in empirical studies—may play an important role in explaining variation among populations and species. Furthermore, we found no evidence that within-season divorce and extra-pair mating are complementary strategies. We discuss our findings within the current evidence of the adaptiveness of secondary mating strategies and their interplay that ultimately shapes the evolution of social monogamy.

## 1. Introduction

In the majority of socially monogamous bird species, two adults form a social bond (i.e. a pair bond) and provide care to the offspring [1]. Pair bonds that are maintained over subsequent breeding seasons are important contributors to fitness [2–5]. However, social, cognitive and temporal constraints on mate choice can result in suboptimal partnerships [6,7]. To correct for a suboptimal partnership, an individual may switch a social partner (anthropogenically termed ‘divorce’, [8,9]), potentially gaining both direct benefits (e.g. increased parental care) and indirect benefits (improved genetic make-up of the offspring). Comprehensive meta-analyses showed that divorce between seasons is commonly triggered by relatively low breeding success and that divorce generally leads to an increase in breeding success (although for females only [10,11]). These findings indicate that between-season divorce is an adaptive behavioural strategy across monogamous birds (although non-adaptive explanations are difficult to exclude for some studies). Social partner change may also occur within a breeding season. Compared to between-season divorce, individuals divorcing within a season are likely to face additional constraints, such as social (any candidate partners might be paired already), or temporal (limited time to find a partner) constraints. These constraints will be particularly strong when the breeding season is short [7,8], which is typical for single brooded populations (where second clutches are initiated only if the first brood fails). Within-season divorce has been studied much less than between-season divorce, and it remains unknown whether it is a secondary mating strategy aimed at improving breeding success.

Divorce may not always be the best or even possible secondary mating strategy. For example, sometimes it will lead to low breeding success [12,13], or even death [4,5,14]. Such negative effects (or constraints) may be avoided by mating outside the social pair bond (i.e. extra-pair mating), although extra-pair paternity (EPP) generally only provides indirect genetic benefits to the offspring and no direct benefits to the female (but see [15–17]). Species with high between-season divorce rates were found to have high EPP [18], supporting the hypothesis that the two are complementary secondary mating strategies and that the rates of both behaviours are higher in species with larger variation in mate quality. However, such a pattern can also be explained if species with strong pair bonds rarely divorce, while for those with very weak bonds the actual mate might be relatively unimportant compared to other factors (e.g. habitat quality). A more recent study based on a larger sample size found no evidence for an among species association between EPP and between-season divorce [19].

Here, we first use a meta-analysis approach [20,21] to weigh the evidence for the adaptiveness of within-season divorce as a behavioural strategy aimed at increasing breeding success. We do this by testing several hypotheses related to breeding success before and after divorce. We could not study fitness effects beyond those of the first post-divorce attempt, since this is what the empirical studies typically focussed on. We then use a meta-regression to test whether EPP or other biological and methodological moderators influence the strength of the association between breeding success and within-season divorce. We evaluate our findings in light of the knowledge on the role of secondary mating strategies and their interplay in socially monogamous species.

## 2. Hypotheses and predictions

Based on the published literature we tested the following hypotheses.

### (a) Within-season divorce is an adaptive behavioural strategy

If within-season divorce is an adaptive strategy to correct for sub-optimal partnership then we expect that:

**Prediction 1i:** Divorce is triggered by low breeding success: pairs that will divorce to their next breeding attempt have lower breeding success in the current attempt compared to pairs that will stay faithful or where one partner dies.

**Prediction 1ii:** Divorcing birds have higher breeding success with their new partner compared to widowed birds, but not necessarily when compared to faithful birds (see [10]). This is because any partner change (widowhood, divorce) likely brings associated costs (e.g. re-adjusting parental effort with the new partner). Thus, even if divorce is adaptive for some individuals, they are still likely to do worse with their new partner compared to faithful individuals who keep the same, familiar partner. However, divorced birds should do better than widowed birds because they traded-off the previous for a better partner, while widowed birds did not make the active choice of leaving the previous partner. Likewise, we predict that the improvement in breeding success (i.e. between two subsequent breeding attempts) is higher for divorced compared to widowed birds, but not necessarily compared to faithful birds. Unfortunately, we were not able to test the latter prediction as our systematic review identified only two studies (with three effect sizes) that recorded the change in breeding success. Further, no study examined breeding consequences beyond a single breeding season, although divorce might have long-term fitness consequences (see in [10]).

### (b) Earlier components of breeding success are the main triggers of divorce

Previous meta-analysis detected that earlier components of breeding success (lay date, clutch) rather than later ones (at hatching and fledging level) were the main triggers of between-season divorce [10]. A possible explanation for the finding is that the effects of environmental stochasticity (e.g. food abundance [22]) accumulate over the breeding cycle. Thus, breeding failure at later stages will increasingly depend on this stochasticity, and proportionally less on parental quality. Results from a meta-analysis [23] support the idea that early components of fitness are indicators of male quality: female's investment in the clutch is based on male quality. However, we acknowledge there could be other (non-adaptive) explanations (e.g. change of territory, [10]). Thus Prediction 2 is that the effect size of the relationship between pre-divorce breeding success and divorce is larger for earlier components of breeding success.

### (c) Females benefit from divorce more than males

Culina *et al*. [10] found that females improved their breeding success via between-season divorce, while males did not. This finding was in line with the hypothesis that females might be initiators of divorce in monogamous birds [8]. We expect to find a similar pattern for within-season divorce (Prediction 3). Since the set of studies identified via our systematic review (see Methods) detected too few studies that report on change in breeding success, we had to limit our analyses to compare breeding success of females and males post-divorce only.

### (d) Benefits of divorce vary with rates of EPP

Divorce and EPP are commonly considered as alternative or complementary strategies that serve a common purpose [18], i.e. correcting for suboptimal partnership. We examine whether the benefits of within-season divorce are associated with levels of EPP. If we assume that EPP is not constrained by external factors (thus high rates reflect high benefits), then a positive association would indicate EPP and divorce can be considered complementary strategies. By contrast, a negative association would indicate that when EPP is constrained, divorce can be used as an alternative strategy serving the same purpose.

## 3. Methods

We performed a systematic review and meta-analyses of eligible studies. A study was eligible if it:

(1) was conducted on a predominantly socially monogamous species with bi-parental care. We determent the level of social monogamy based on information on the species (or a population) as provided in the corresponding study retrieved by our systematic review;
(2) either compared breeding success between (a) divorced and widowed, divorced and faithful or widowed and faithful pairs/individuals in one of the following: (i) the first breeding attempt of the season, (ii) the second breeding attempt of the season, (iii) the change of breeding success between the two attempts; or (b) breeding success (change in breeding success between two attempts, or breeding success in the second attempt) between divorced females and males, or between widowed females and males.

### (a) Literature search

We conducted a systematic literature search for studies published in English via 15 online databases/search platforms (see electronic supplementary material, Methods) in March 2022. The search syntax included three main components that were designed to describe: (a) pair-bond dynamics and renesting (e.g. ‘mate change’ OR renest × OR ‘mate retention’); (b) breeding success (e.g. ‘breeding success’ OR ‘breeding output’); (c) that breeding attempts were within the same breeding season (e.g. ‘within the season’ OR ‘within a year*’). The syntax was adjusted according to the search functionality of each platform: the details on the complete syntaxes (used for each database) can be found in the electronic supplementary materials, Methods. Following the steps of Preferred Reporting Items for Systematic Review and Meta-analysis (PRISMA, [24]) 33 eligible studies were detected (see data tables, also electronic supplementary material, Methods for PRISMA diagrams). One study was further excluded because it was the only one measuring lifetime reproductive success, and one because it only measured change in breeding success (see electronic supplementary material, Methods).

### (b) Calculating effect sizes

We converted all the effect sizes into *r*, and then into Fisher's *Z*_r_ for normality, with accompanying SEs (formulas in [25]). The exact formulas can be found in electronic supplementary material, Methods. We assigned a positive direction to the effect size if it implied that divorce is an adaptive strategy (see ‘Hypotheses and predictions' section). For effect sizes that compared the success among divorced males and females, we assigned the biological direction to the effect size to be positive if divorce was adaptive for females (this being an arbitrary choice but following Prediction 3). In a few instances separate effect sizes from the same study were combined (e.g. if a study reported values for separate years these were combined, see electronic supplementary material, Methods for details).

We constructed separate datasets (table 1), each for one of the three meta-analyses, with effect sizes that:

(1) relate breeding success in the breeding attempt before divorce (*t* − 1) to the occurrence of divorce (*before meta-analysis*) to test Prediction 1i and Prediction 2.
(2) relate divorce to the breeding success in the breeding attempt after divorce (*t*) (*after meta-analysis*) to test Prediction 1ii.
(3) examine breeding success consequences of divorce between males and females (*FvsM meta-analysis*) to test Prediction 3.

**Table 1.** Sample size (*N* of effect sizes, species, studies) and references for each of the three meta-analyses on within-season divorce and breeding success.

analysis	*N* of effect sizes	*N* of species	*N* of studies	references
before	27	10	13	[26–38]
after	43	14	16	[28,34–48]
FvsM	20	9	10	[35,45,48–55]

To obtain the effect sizes for the *FvsM meta-analysis* we used the effect sizes that originally compared divorced or widowed males to divorced or widowed females, and those where data (i.e. means and s.d. of breeding success) was reported separately for divorced/widowed females and males (but where the original study itself did not compare breeding success between the sexes).

### (c) Meta-analysis and meta-regression

We ran three separate sets of multi-level meta-analyses and meta-regressions (*before*, *after*, and *FvsM*). All the analyses were implemented in package ‘MCMCglmm’ v. 2.32 [56] in R v. 4.0.4 [57]. To incorporate effect sizes that come from the same species (and to account for phylogenetic relatedness between species) or study we used a multi-level model. Phylogenetic trees were constructed using birdtree.org [58] and implemented via package ‘ape’ v. 5.5 [59]. As the majority of species in any of the datasets was represented by a single study, species and study identity are largely confounded. Thus, two sets of analyses were run for each of the three meta-analyses—one accounting for phylogeny only and one accounting for study ID, by including phylogeny or study ID as a random intercept. The results obtained by these two sets of analyses did not qualitatively differ. In the main text, we present the estimates obtained by the random-effect model that returned the lowest DIC value (‘phylogeny’ for *before* and *after meta-analyses*, ‘study’ for *FvsM meta-analysis*). To account for uncertainty in phylogenetic trees, all the models were rerun 100 times (each time using a different tree) with no qualitative difference in the results.

We first tested if any of the methodological moderators (one at the time) influenced the meta-analytic mean. A moderator was included only if each level of the moderator had 10 or more data points. Thus, not all of the moderators were tested in each meta-regression. Methodological moderators included: (i) type of study (in *after* and *FvsM meta-analyses*): experimental or observational because in contrast to the general pattern across species [10], experimental studies have found no evidence that reproductive failure would trigger divorce (e.g. [60]) (ii) whether the measure of breeding success was dichotomized (i.e. divided into binary categories, for example, hatchlings produced versus no hatchlings produced) or not (*before meta-analysis*); (iii) whether renesting occurs after the failure of the first nest: no, yes, and sometimes. Populations that only renest after breeding failure are generally single brooded, while double-brooded populations may renest after success and sometimes after failure. In the *before* dataset, all effect sizes come from multi-brooded populations (with values ‘no’ and ‘sometimes’), whereas in the *after* dataset we combined values ‘no’ and ‘sometimes’ due to limited sample sizes of the ‘no’ category; (iv) and comparison class (*FvsM meta-analysis)*, which coded for whether the effect size contrasted widowed females and males, or divorced females and males.

We then modelled the influence of the breeding success component on the effect size. Since few studies examined the role of lay date we were unable to include this component. Further, low sample sizes prevented us from examining each component of breeding success separately. Thus, in the *before meta-analysis* breeding success was (post-data collection) defined as brood level (clutch size, hatching success—either a binary variable or a percentage of hatched eggs, brood size) or fledging level (number of fledglings, fledging success—either a binary variable or a percentage of fledged nestlings, number of offspring alive on day 11 post-hatch multiplied by the mean mass of nestlings in the brood). In the *after meta-analysis* these were: renesting (time for a bird to renest), brood level (as above), and fledgling level (as above). In the *FvsM meta-analysis* these were: renesting (whether a bird has re-mated, time to re-mate, whether a bird had renested, time to renest) and breeding success (any of the breeding success measures).

To test whether EPP rates explain variation in effect sizes we added estimates of EPP rate (as reported in [61], based on the percentage of broods with at least one EP offspring) to the best-supported random-effect model for each meta-analysis (*before*, *after*, *FvsM*). Here, we used subsets of the original effect sizes confined to species with known EPP rates (see data tables).

For each meta-analysis and meta-regression, we used inverse-gamma priors (*V* = 1, nu = 0.002). Models were run with 2 million iterations (nitt), thinning intervals of 1000 (thin) and burn-in of 200 000. Model selection was based on the deviance information criterion (DIC, Bayesian equivalent of AIC) and credible intervals (CrI) for the moderator. DIC relies on posterior distributions to approximate normality, and we visually inspected the posterior distributions for non-normality.

**Figure 1.**
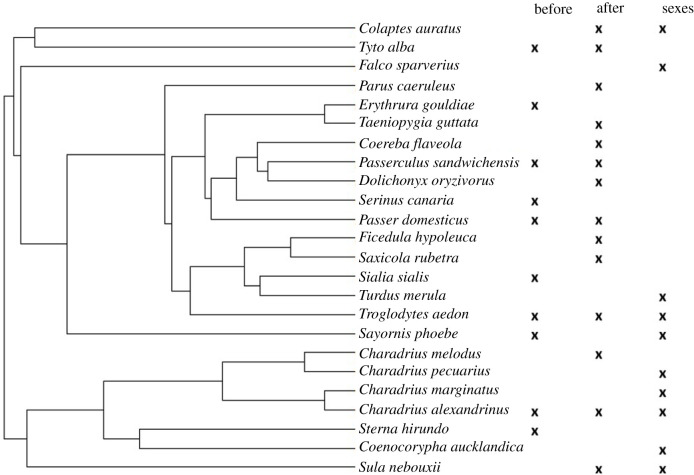
Phylogenetic tree of species included in the three meta-analyses on (1) breeding success before divorce and occurrence of within-season divorce; (2) within-season divorce and breeding success after divorce; (3) breeding success compared between divorced/widowed females and males. The right-hand side of the figure indicates species included in each meta-analysis.

We calculated total heterogeneity, and heterogeneity due to study or species phylogenetic effects following procedures outlined in [62] figure 1.

### (d) Publication bias

To check for time-lag bias [63,64] we included publication year as a moderator to the best supported random effect model. Small study effect bias was checked for based on Eggers regression where the effect size is regressed on the effect standard error [65]. To allow for the non-independent data points, we have used Egger's regression test with the model structure of the best-supported model in each dataset in MCMCglmm with the S.E. added as a covariate. Further, we estimated the number of missing studies based on trim-and-fill analysis [66]. We conducted trim-and-fill using random-effect meta-analysis with the restricted maximum-likelihood estimation (REML) in the R package ‘metafor’ v. 2.4.0 [67]. We did this for each of the three datasets, using all the effect sizes, or using one randomly chosen effect size per study and repeating the trim-and-fill procedure 2000 times.

## 4. Results

Our full dataset included 31 relevant studies on 24 socially monogamous bird species (table 1 for summary, and data tables), belonging to six bird orders (15 to Passerifomes; five to Charadriiformes, one each to Falconifomes, Piciformes, Strigifomes and Suliformes). Individuals from socially monogamous species included in our dataset were more likely to divorce within a season (mean proportion of individuals divorcing = 0.25, s.d. = 0.23, range 0–0.70, based on 26 values from 16 species) than to engage in EPP (mean percentage of broods with at least one EP young = 0.16, s.d. = 0.19, range 0–0.65, based on 18 values from 18 species, [61]).

Total heterogeneity in effect sizes $\left(I_{\mathrm{total}}^{2}\right)$ following [68] was estimated to be moderate (67% in *FvsM* dataset), to high (90% in *before*, 88% in *after* dataset). Part of this heterogeneity was attributed to species phylogeny (26% in *before*, and 80% in *after* dataset, electronic supplementary material, tables S1 and S5) and study (18% in *FvsM* dataset, electronic supplementary material, table S9). No time-lag bias was detected in any of the datasets (electronic supplementary material, table S13). The trim-and-fill analysis detected zero missing studies for the *before* and *FvsM* datasets, and six missing on the right side for the *after* dataset (details in electronic supplementary material, Results, including electronic supplementary material, figure S1 with funnel plots).

**Figure 2.**
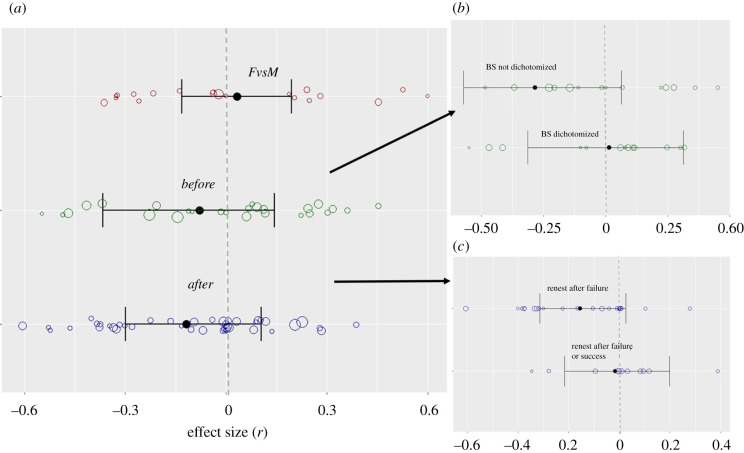
Forest plots of the meta-analytic means (black dots) with 95% CrI (horizontal lines) for the relationship between divorce and breeding success for (*a*) each main meta-analysis: breeding success compared between males and females (FvsM), measured before divorce (before) and after divorce (after); (*b*) dichotomized versus non-dichotomized effect sizes for before dataset; (*c*) populations that have replacement clutches (renest only after failure) and multi-brooded populations, in the *after* dataset. Positive values of the effect sizes are those that support divorce being adaptive in monogamous birds (*before* and *after* dataset), and divorce as a female-driven strategy (females benefit from divorce more than males, *FvsM* dataset). Open circles represent effect sizes as calculated from each primary study with their size proportional to log(sample size).

By contrast to Prediction 1, we did not find evidence that within-season divorce is triggered by low breeding success, rather, estimates of the best meta-analytic model indicated that divorced birds had slightly higher breeding success before divorce compared to widowed and faithful birds (*r*_before_ = −0.079; 95% CrI: −0.366/0.144, figure 2*a*). Divorced birds also had lower post-divorce success (*r*_after_ = −0.118; 95% CrI: −0.299/0.105) compared to other groups (figure 2*a*). Prediction 2 (earlier components of breeding success are main triggers of divorce) was also not supported: the model distinguishing which measure of breeding success was used (brood or fledging level) received less support than the intercept-only model (ΔDIC = 3.29, electronic supplementary material, table S3).

The global meta-analytic mean (of the random effect model) of the *FvsM* dataset was positive but small (*r*_FvsM_ = 0.033, 95% CrI: −0.132/0.198, figure 2*a*), meaning that divorced and widowed females might be doing slightly better than their male counterparts. Further, the model with the breeding success component (whether a bird renested at all and how soon versus its breeding success) gained better support than the intercept-only model (7.3 lower DIC, electronic supplementary material, table S11). The direction of the meta-analytic mean showed that divorced and widowed females might renest sooner and more frequently than divorced males (*r* = 0.115, CrI: −0.089/0.297, electronic supplementary material, table S11), but have lower breeding success (*r* = −169, CrI: −0.427/0.110). Thus, Prediction 3 was also not supported.

Including EPP rates did not improve the model fit compared to the intercept-only model in any of the meta-analyses (electronic supplementary material, tables S4, S8 and S12).

Modelling of the methodological moderators showed that ‘dichotomization’ (i.e. whether or not measure of breeding success was dichotomized) in the *before* meta-analysis improved the model fit (DIC lover by approximately 47 units, electronic supplementary material, table S2). This model estimated a negative meta-analytic mean for non- dichotomized breeding success (*r* = −0.285, CrI: −0.573/0.063, figure 2*b*), suggesting that higher success triggers divorce (but note the large CrIs). Including the moderator that reflects whether a population had replacement clutches rather than being a multi-brooded one in the *after* meta-analysis, showed that divorced individuals of single-brooded populations did worse than faithful and widowed individuals (*r* = −0.155, CrI: −0.315/0.025, figure 2*c*).

There was no evidence (based on model selection) that any other moderator we considered influences the effect sizes (e.g. experimental studies did not differ from observational studies in either the after and the FvsM datasets, electronic supplementary material, tables S6 and S10).

## 5. Discussion

Divorce and extra-pair mating are often viewed as secondary mating strategies aimed at correcting for suboptimal partnerships. Divorcing between breeding seasons appears to generally be adaptive across species [10]. On the contrary, our meta-analyses based on the overall sample of 90 effect sizes from 24 socially monogamous bird species with bi-parental care did not provide evidence that within-season divorce is an adaptive behavioural strategy. Further, fitness benefits/costs of divorce did not vary with the rate of EPP. However, some of our estimates are indicative of existing trends. Below we evaluate our findings and discuss these in the light of the current knowledge on the secondary mating strategies and their interplay. We also discuss the limitations of our meta-analyses.

### (a) No evidence for the adaptiveness of within-season divorce

Our meta-analyses showed that, across socially monogamous birds, within-season divorce was not triggered by low breeding success, nor did divorced birds have higher breeding success in their second attempt (i.e. post-divorce) compared to faithful or widowed birds. Further, 20 effect sizes on nine species failed to provide evidence for a difference in the fitness consequences of divorce between males and females. Interestingly, although the meta-analytic mean for the effect sizes of breeding success preceding within-season divorce was in the opposite direction than the meta-analytic mean of between-season divorce [10], the impact of divorce on breeding success immediately after within-season divorce is very similar (−0.118; 95% CrI: –0.299–0.105) to the previously reported estimate on between-season divorce (−0.110, 95% CrI: –0.191/–0.031, [10]). This suggests that the resulting immediate costs might be similar for within- and between-season divorce, but that the power to detect significant effect for within-season divorce is reduced because of the much smaller dataset (34 versus 128 effect sizes in *after* dataset) and large heterogeneity in effect sizes. A shortcoming of the current literature on within- and between-season divorce is the focus on the immediate fitness consequences of divorce. While any partner change (widowhood, divorce) entails costs (e.g. lower coordination between new pairs, inexperience with the breeding site for at least one of the partners [69–71]), these initial costs might be compensated later. For example, in experimentally induced remating Eurasian oystercatchers (*Haematopus ostralegus*) newly formed pairs initially performed poorly, but were able to advance their lay date (a strong indicator of breeding success) over the next 4 years [3]. In addition, divorce may result in fitness benefits other than the quantity of offspring (used in the empirical studies in our dataset). For example, if divorce is the result of (genetic) incompatibility between partners, re-mating might result in higher quality rather than quantity of offspring [72].

While some populations are mutlibrooded, in other populations second (i.e. replacement) clutches are only laid if the first breeding attempt fails. Although all populations in the *before* dataset were multi-brooded, examining the influence of multi- versus single-broodedness in the *after* meta-analysis showed that divorced individuals had lower breeding success compared to widowed and faithful individuals in single-brooded, but not multi-brooded, populations (figure 2*c*). An explanation for this could be that compared to multi-brooded populations, in single-brooded populations time to renest and find a new mate is likely more limited. Further, whether or not individuals have multiple broods might vary systematically among individuals within the same population. For example, earlier lay date (e.g. [73]), and age [74]—which are often taken as indicators of an individual's quality—affect the likelihood that individuals will breed multiple times in a season. This likely creates a bias to specific individuals within a population that breed multiply (either with the same or with a different partner) and that might share a common trait (e.g. quality) that allows them to breed multiply. This shared trait could mask any benefits of a specific within-season mating strategy.

Finally, heterogeneity in effect sizes was large, particularly in the *before* (90%) and *after* (88%) datasets, and many of the credibility intervals of the meta-analytic means overlapped zero. Phylogenetic effects explained a large part of heterogeneity in the *after* meta-analysis*.* Thus, within-season divorce might be beneficial for breeding success in some species (e.g. *Tyto alba*) but costly in others (e.g. *Sula nebuloxii,* see data tables for estimates). Social, demographic and environmental factors also likely differ between species, and between populations of the same species, altering the costs and benefits of within-season divorce. Unfortunately, many studies (included in this, but also in the previous meta-analyses on between-season divorce [10,11]) did not provide sufficient information on these factors for analyses, nor did they control for other factors that can influence both divorce and breeding success (e.g. age or the experience of partners).

### (b) The interplay between divorce and EPP

We did not find that the benefits of divorce varied with levels of EPP, and thus did not find evidence in support of these behaviours being complementary or alternative strategies serving the same purpose (i.e. to obtain a genetically better quality or more genetically compatible mate). The absence of any association could have many reasons, but two potential explanations are that divorce and EPP are strategies with a different purpose, or that (one of) the behaviours are non-adaptive.

The most obvious difference between EPP and divorce is the type of fitness consequences. Through mating extra-pair, males can increase their breeding success and females can gain genetic benefits for the offspring (e.g. [75,76]). Divorce and re-mating however, may result in (additional) social benefits for example through improved parental care or territory defence behaviours [77]. Costs may also vary in various ways. First, seeking EPP may result in retaliation by the pair male and reduced male care when he loses confidence in paternity [78,79], as supported by meta-analysis across monogamous species [80]. Divorce and re-mating on the other hand bear potential costs of not obtaining the new partner at all (and thus skipping breeding) or losing any benefits of mate familiarity [81,82]. Second, while searching for an EP mate may be costly as it trades off with breeding duties like paternal care or mate-guarding [75,83,84], searching for available (but limited) social mates will trade off with behaviors like predator vigilance or feeding (in the period between breeding attempts). The expected timescale of the costs and benefits will also vary between divorce and EPP. Re-mating after divorce will be longer-term, while EPP typically only involves a single breeding event (although studies have shown that females may consistently mate with the same extra-pair male, [85,86]).

Altogether, EPP and divorce will likely have very different consequences for current and future fitness. Thus, we argue that across species these behaviours cannot be seen as alternative or complementary strategies serving the same purpose [18]. More likely, EPP and divorce have different purposes, that may partly overlap in some, but not other species.

Alternatively, and in contrast to the commonly assumed adaptive explanations, one or both of these secondary mating strategies might be non-adaptive and vary because of other proximate mechanisms. Although between-season divorce seems to be an adaptive secondary mating strategy across socially monogamous species ([10], albeit lacking experimental studies), this is not the case for within-season divorce (this study). Furthermore, whereas the benefits of EPP are clear for males and some studies have shown that females can gain benefits in some species [87–89], many studies failed to detect the benefits of EPP for females [61] leading to its adaptiveness overall being questioned [90]. Thus, variation in divorce and EPP might simply exist because of covariation with other traits. For example, it has been suggested that extra-pair behaviour can evolve via indirect selection on males [91,92], however convincing emprical support for this idea is lacking [93,94].

Lastly, a current limitation to our study is that similarly to divorce [4,5], EPP rates can vary substantially among populations of the same species [62]. Additionally, population-level patterns rarely provide comprehensive insights into processes happening at the individual level and at the finer timescale. Thus, rather than using a species estimate of the EPP rates, it would be preferable to use data on EPP, divorce, and fitness that has been collected simultaneously, within populations, and for individuals. Interestingly, species in our dataset were on the lower spectrum of the overall range of the EPP rates detected in socially monogamous species: 16% versus 25% on average for socially monogamous birds [61]. We can only speculate about this pattern, as unfortunately, few studies examined EPP and divorce simultaneously (but see e.g. [95]).

### (c) Outlook for future studies

Our study provided no evidence for the adaptiveness of within-season divorce, with the large heterogeneity in effect sizes and the strong phylogenetic signal suggesting phylogenetic, social, and environmental factors may play an important role in explaining variation among populations and species. In addition to broader sampling across the avian tree, examining the functional interplay between secondary mating strategies is only fully informative when data on both divorce and EPP are collected simultaneously, through detailed individual-based studies. There is also an urgent need for more experimental studies to exclude potential bias due to external factors or to individual ‘quality’. For example, preventing individuals from engaging in EPP [96] could shed light on whether this results in increased divorce rates. Finally, examining the fitness costs and benefits beyond the immediate consequences following divorce will prove crucial in understanding the adaptiveness and evolution of secondary mating strategies.

## Data Availability

Data sets associated with this article and R codes to conduct the meta-analyses and produce main text and electronic supplementary material figures can be found at Dryad Digital Repository: https://doi.org/10.5061/dryad.3n5tb2rkq [97]. Please read the README file for the description of the data and code files. Additional information is provided in electronic supplementary material [98].
